# Olfactory receptor Olfr78 (prostate-specific G protein-coupled receptor PSGR) expression in arterioles supplying skeletal and cardiac muscles and in arterioles feeding some murine organs

**DOI:** 10.1007/s00418-021-02032-6

**Published:** 2021-09-20

**Authors:** Petra Mermer, Jörg Strotmann, Wolfgang Kummer, Renate Paddenberg

**Affiliations:** 1 Institute of Anatomy and Cell Biology, Excellence Cluster Cardio-Pulmonary Institute, German Center for Lung Research, Justus Liebig University, Aulweg 123, 35385 Giessen, Germany; 2 Institute of Physiology, University of Hohenheim, 70599 Stuttgart, Germany

**Keywords:** Arterioles, Mouse, Olfactory receptor Olfr78, Prostate-specific G protein-coupled receptor PSGR, Striated muscles, X-gal staining

## Abstract

The olfactory receptor Olfr78 (prostate-specific G protein-coupled receptor PSGR) is a member of the G protein-coupled receptor family mediating olfactory chemosensation, but it is additionally expressed in other tissues. Olfr78 expressed in kidney participates in blood pressure regulation, and in prostate it plays a role in the development of cancer. We here screened many organs/tissues of transgenic mice co-expressing *β*-galactosidase with Olfr78. X-gal-positive cells were detectable in smooth muscle cells of numerous arterioles of striated muscles (heart ventricles and skeletal muscles of various embryological origin). In addition, in most organs where we found expression of Olfr78 mRNA, X-gal staining was restricted to smooth muscle cells of small blood vessels. The dominant expression of Olfr78 in arteriolar smooth muscle cells supports the concept of an important role in blood pressure regulation and suggests a participation in the fine tuning of blood supply especially of striated muscles. This should be considered when targeting Olfr78 in other contexts such as prostate cancer.

## Introduction

G protein-coupled receptors (GPCRs) are important for many functions in the human body including signalling in vision and smell, regulation of mood and behaviour, the nervous and the immune system, inflammation, and many other biological processes. Therefore, it is not surprising that GPCRs represent the largest family of protein targets for approved drugs (Sriram and Insel 2018; Heifetz et al. 2015).

Olfactory receptor (OR) Olfr78 is a member of the GPCR family and mediates olfactory chemosensation in the nose. Unfortunately, several different names are used for Olfr78, depending on the species analysed and the organs investigated. Olfr78 [= official name according to the Mouse Genome Informatics (MGI); aliases: 4633402A21Rik, MOL2.3, MOR18-2, Or51e2, PSGR, RA1c] was first discovered to be highly expressed in human prostate and thus initially named prostate-specific G protein coupled receptor (PSGR). It was later found that human PSGR belongs to the OR superfamily; hence, it was renamed as OR family 51 subfamily E member 2 (OR51E2) [= official name according to the HUGO Gene Nomenclature Committee (HGNC); aliases: PSGR, HPRAJ, OR52A2, OR51E3P]. However, in the community working on prostate, PSGR is still a commonly used term for murine models and PSGR or OR51E2 for human studies. In rat, Olfr78 is called Olr59 [according to the Rat Genome Nomenclature Committee (RGNC); aliases: Olfr78, Or51e2, RA1c].

Particularly through transcriptomic research, Olfr78 was found to be expressed not only in the olfactory epithelium but also in many other tissues including kidney and prostate. Pluznick et al. (2013) used a mouse strain in which the gene encoding Olfr78 was replaced by *β*-galactosidase and found expression of Olfr78 in smooth muscle cells of small resistance vessels and in juxtaglomerular arterioles of the kidney. They identified the short chain fatty acids (SCFA) acetate and propionate as ligands of Olfr78 and demonstrated the participation of Olfr78 in blood pressure regulation. For this reason, the receptor and its ligands may be relevant in the pathogenesis of hypertension and development of new drugs for treatment of this disease.

OR51E2 is expressed in prostate epithelial cells, and expression is significantly increased in prostate intraepithelial neoplasia and prostate cancer relative to healthy tissue and benign prostatic hyperplasia (Weng et al. 2005). Although the exact function of the receptor in prostate cancer has not yet been clarified, these data indicate the importance of OR51E2 in the pathogenesis of prostate cancer and OR51E2, and its ligand may be a new target to better combat prostate cancer.

Against the background of the potential importance of Olfr78 for the development and treatment of serious diseases, the aim of our work was to examine the expression of Olfr78 in mouse. Identification of Olfr78 protein-expressing cells may point to further functions of the receptor. We performed RT-PCR to analyse Olfr78 mRNA expression and used reporter mice co-expressing *β*-galactosidase and Olfr78 to detect Olfr78-expressing cells in several murine organs and tissues.

## Materials and methods

### Animals

In this study, we used C57Bl6 wildtype (WT) mice (*n *= 3) and the transgenic mouse line MOL2.3-IGITL (designation in our animal facility and in the permission: CD1;129-Olfr78^tm1jgst^) (*n *= 17). In this mouse line, green fluorescent protein (GFP) and tau/*β*-galactosidase are co-expressed with Olfr78 (Conzelmann et al. 2000). Mice of both sexes aged between 2 and 11 months were used.

Mice were housed in GM 500 individually ventilated cages (Tecniplast Deutschland GmbH, Hohenpeißenberg, Germany) under specific pathogen-free conditions at 23 °C temperature and 50% humidity. Mice had free access to water and standard diet pellets (Altromin Spezialfutter GmbH & Co. KG, Lage, Germany). They were kept in 14 h light/10 h dark cycle conditions. Each cage was equipped with a mouse house and rodent wood as environmental enrichment. The cage-change interval was about 7 days.

All experiments were performed according to the national and international guidelines and with approval of local authorities (Animal Welfare Officer at the University of Giessen) and of the Federal Authorities for Animal Research of the Regierungspräsidium Giessen (Hessen, Germany), registration number (no. 614_M).

### Reverse transcription-PCR (RT-PCR)

Total RNA from organs/tissues [carotid bifurcation together with the superior cervical ganglion and attached carotid body, olfactory mucosa, tip of the tongue, outer ocular muscles, cheek muscles, thigh muscles, diaphragm, intercostal muscles, heart, trachea, lung, oesophagus, stomach, small intestine, colon, liver, gallbladder, kidney, urinary bladder, adrenal gland, thymus, spleen, lymph node, testis, epididymis, prostate, seminal vesicle, ovary, oviduct, uterus, vagina, femoral artery, vein and nerve, adipose tissue, auricle of the ear, and DRGs (dorsal root ganglia); *n *= 1–5 for each organ/tissue; exact numbers are given in Table 1] was extracted using RNeasy Micro Kit (Qiagen, Hilden, Germany) according to the manufacturer’s protocol. For removal of genomic DNA contamination, up to 1 μg RNA in 8 μl water was incubated with 1 μl 10 × DNase reaction buffer and 1 μl DNase (1 U/μl; Invitrogen, Darmstadt, Germany) for 15 min at 25 °C. Then, 1 μl ethylenediaminetetraacetic acid (25 mM) was added to each sample. After 10 min incubation at 65 °C, samples were rapidly chilled on ice and 9 μl reaction mixture containing 1 μl oligo-dT (50 μM), 1 μl dNTPs (10 mM), 1 μl Superscript RNase H- Reverse Transcriptase (RT; 200 U/μl), 4 μl 5 × first-strand buffer, and 2 μl dithiothreitol (0.1 M; all reagents were purchased from Invitrogen, except dNTPs, which were from Qiagen) was added to each sample and incubated for 50 min at 42 °C and 10 min at 70 °C. PCR was performed with cDNA samples using the following protocol: 2 μl cDNA as template, 2 μl MgCl_2_ (25 mM), 2.5 μl 10 × PCR buffer II, 0.75 μl dNTPs (10 mM), 0.75 μl of each primer (10 μM), 0.25 μl AmpliTaq Gold DNA Polymerase (5 U/μl; all reagents were obtained from Applied Biosystems, Darmstadt/Germany), and 16.75 μl H_2_O. The following cycling conditions were chosen: initial denaturation at 95 °C for 12 min, followed by 39 cycles of 30 s at 95 °C, 30 s at 60 °C, 30 s at 72 °C, and a final extension at 72 °C for 7 min. Controls without RT were performed for colon, olfactory mucosa, vagina, and diaphragm. The primers used in the study were for Olfr78: fwd: CCT GCT GCT CTC CTT GAG TA; rev: AGT GAG CTT CCT CCA GTC CT; product length: 159 bp and for β-actin: fwd: GTG GGA ATG GGT CAG AAG G; rev: GGC ATA CAG GGA CAG CAC A; product length: 300 bp. Control reactions were run in the absence of reverse transcriptase and in the absence of DNA template, respectively. The PCR products were separated by electrophoresis on a 2% Tris-acetate-EDTA agarose gel. For subsequent sequencing, PCR products were purified using the Monarch DNA Gel Extraction Kit (New England Biolabs, Frankfurt, Germany) as recommended by the manufacturer. Sequencing was done by Eurofins (Ebersberg, Germany).

**Table 1:** Analysis of Olfr78 mRNA expression and X-gal staining in murine organs

Organ/tissue	Olfr78-positive samples/total *β*-actin-positive samples	Localisation of X-gal staining
Superior cervical ganglion + carotid body	5/5	Glomus cells of the carotid body
Olfactory mucosa	5/5	Some olfactory neurons
Tongue	4/5	Small vessels
Outer ocular muscle	5/5	Small vessels
Cheek muscle	4/4	Small vessels
Thigh muscle	1/2	Small vessels
Diaphragm	5/5	Small vessels
Intercostal muscle	1/1	Small vessels
Heart ventricles	3/4	Small vessels
Atrial auricles	5/5	Almost completely neg
Apex cordis	3/3	Small vessels
Trachea	1/1	Very few small vessels
Lung	2/5	Negative
Oesophagus	1/1	Very few small vessels
Stomach	4/5	Very few small vessels (not shown)
Small intestine	1/5	Very few small vessels (not shown)
Colon	5/5	Some small vessels + enteroendocrine cells
Liver	1/5	Negative
Gallbladder	4/5	Cystic artery
Kidney	2/5	Specific vascular segments
Urinary bladder	5/5	Very few small vessels
Adrenal gland	5/5	Negative
Thymus	4/5	Negative (not shown)
Spleen	1/4	Negative
Lymph node	1/2	Not analysed
Testis	2/2	Small vessels and maturing sperm
Epididymis	2/2	Some small vessels
Prostate	2/2	Not analysed due to high endogenous X-gal activity
Seminal vesicle	0/1	Negative
Ovary	3/3	Negative
Oviduct	3/3	Some small vessels
Uterine horn	3/3	Some small vessels
Uterine fundus	3/3	Small vessels
Vagina	2/3	Some small vessels (not shown)
Adipose tissue	1/1	Some small vessels
Dorsal root ganglia	0/1	Negative
Femoral artery	3/5	Positive smooth muscle cells
Femoral vein	1/5	Negative
Femoral nerve	0/4	Negative
Auricle of the ear	3/5	Negative

### Whole-mount X-gal staining of mouse organs and tissues

Mice were killed by inhalation of an overdose of isoflurane (Baxter, Unterschleissheim, Germany) and exsanguination. Organs/organ packages and skin-free hind limbs with part of the pelvis were dissected and immediately transferred into 4 °C cold 4% paraformaldehyde (PFA) in 0.1 M phosphate buffer. Samples were kept on ice for 1 h and subsequently washed with 4 °C cold washing buffer [phosphate-buffered saline (PBS), pH 7.4, 2 mM MgCl_2_, 0.01 mM sodium deoxycholate, 0.02% octylphenoxypolyethoxyethanol (IGEPAL CA-630)] followed by two washing steps using the same buffer at room temperature. The washing buffer was replaced by staining solution [0.1 M Tris–HCl pH 7.5, 2 mM MgCl_2_, 0.02% IGEPAL CA-630, 0.01% sodium deoxycholate, 5 mM potassium ferricyanide, 5 mM potassium ferrocyanide, 1 mg/ml 5-bromo-4-chloro-3-indoxyl-*β*-D-galactopyranoside (X-gal; prepared from a 20 mg/ml stock solution in DMSO)] and samples were incubated light protected overnight at room temperature. Next day, samples were washed three times with PBS, pH 7.4. Photographs were taken with a Zoom Stereo Olympus SZ40 microscope equipped with a camera port and a transmitted light base. For this purpose, samples were fixed with fine pins either on the bottom of a delta T-dish covered with a thin layer of transparent Sylgard polymer (Dow Corning, Wiesbaden, Germany) or on pieces of pink dental wax. All the time samples were submerged in PBS.

### Frozen sections of whole-mount X-gal-stained mouse organs and tissues

After taking photographs, whole-mount samples were fixed again with 4% PFA overnight at 4 °C. Fixative was removed by washing with 0.1 M phosphate buffer pH 7.4 (PP) followed by incubations in the same buffer with 9% and 30% sucrose, respectively. Samples were placed in cryo-embedding medium (Tissue-Tek, Sakura, Umkirch, Germany) and shock-frozen in liquid nitrogen. Next, samples were sectioned at 10 μm thickness with a cryostat microtome (Leica CM 1900, Wetzlar, Germany), mounted onto Superfrost Plus glass slides (R. Langenbrinck, Emmendingen, Germany), and air-dried. After embedding in Mowiol 4–88 (pH 8.6; Merck, Darmstadt, Germany) sections were examined under a Zeiss Axioplan 2 microscope (Jena, Germany) and photographs were taken.

Organs (liver, kidney, heart, testis) from one reporter mouse were used for immunohistological localisation of α-smooth muscle actin. We applied FITC-conjugated anti-α-smooth muscle actin antibody [mouse monoclonal antibody clone 1A4; F3777; diluted 1:2.000 in PBS (Sigma-Aldrich, Taufkirchen, Germany)] or Cy3-conjugated anti-α-smooth muscle actin antibody [mouse monoclonal antibody clone 1A4; C6198; diluted 1:1.600 in PBS (Sigma-Aldrich)] to frozen sections for 1 h at room temperature to identify smooth muscle cells. Abcam, a manufacturer that also offers the mouse monoclonal antibody clone 1A4, reports that the antibody was knockout validated (https://www.abcam.com/alpha-smooth-muscle-actin-antibody-1a4-ab7817.html).

### Tissue clearing of whole mounts

In case of two mice, tissue clearing was performed to optimize visualization of X-gal staining following the protocol given by Treweek et al. (2015). Briefly, samples were incubated in A4P0 Hydrogel solution prepared by mixing 20 ml of 40% acrylamide (wt/vol) (Sigma-Aldrich) and 20 ml of 10 × PBS with 160 ml of ice-cold H_2_O. Immediately before use, 500 mg thermoinitiator (2,2ʹ,-azobis [2-(2-imidazolin-2-yl)propane] dihydrochloride) (FUJIFILM Wako Chemicals Europe GmbH, Neuss, Germany) was stirred into the solution. For penetration, samples were incubated with A4P0 hydrogel solution over night at 4 °C. Next day polymerisation of the hydrogel solution was induced by incubating the samples in a 37 °C warm water bath. Subsequently, polymerized hydrogel attached to the surface of the samples was removed and samples were washed with PBS. For elution of lipids, samples were incubated in PBS-8% sodium dodecyl sulphate (Carl Roth, Karlsruhe, Germany), which was replaced daily by fresh solution. Over the coming days, progress in tissue clearing was checked microscopically.

## Results

### Olfr78 mRNA expression in murine organs

**Fig. 1:**
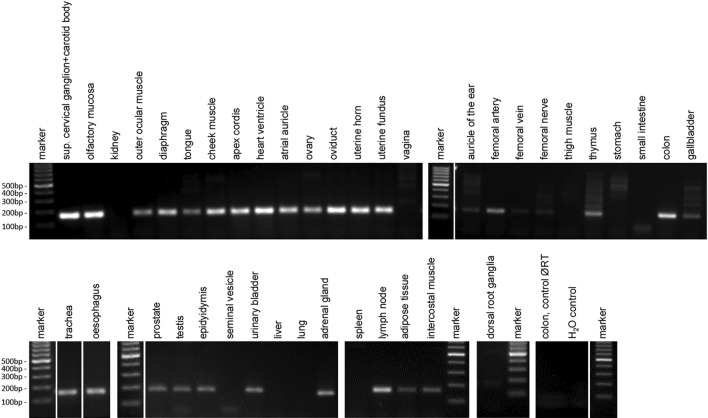
Expression of Olfr78 mRNA transcripts in murine organs. The RT-PCR product was of the expected size and specificity was confirmed by sequencing. For colon, control run without reverse transcriptase (control Ø RT) and control without template (H_2_O control) are shown. The data shown are from organs and tissues isolated from four mice. Individual agarose gels are separated by wide white spaces and samples that were separated on the same gel but not next to each other by narrow white spaces. Marker: 100 bp molecular weight marker

We performed RT-PCR to analyse expression of Olfr78 at the level of mRNA (Fig. 1). *β*-Actin mRNA served as quality control (not shown); only samples for which we obtained a clear *β*-actin PCR product were included in the summary (Table 1). Olfr78 expression was detectable in a large number of murine organs. We obtained PCR products for all samples of superior cervical ganglion with attached carotid body, olfactory mucosa, outer ocular muscle, cheek muscle, diaphragm, intercostal muscle, atrial auricles, apex cordis, trachea, oesophagus, colon, urinary bladder, adrenal gland, testis, epididymis, prostate, ovary, oviduct, uterus, and adipose tissue. Olfr78 mRNA was not in or not in all samples of the following organs detectable: tongue (4/5 = 4 out of 5 samples were positive), thigh muscle (1/2), heart ventricle (3/4), lung (2/5), stomach (4/5), small intestine (1/5), liver (1/5), gallbladder (4/5), spleen (1/4), thymus (4/5), lymph node (1/2), seminal vesicle (0/1), vagina (2/3), auricle of the ear (3/5), DRGs (0/1), femoral vein (1/5), and femoral nerve (0/4). Olfr78 mRNA was expressed in three out of five samples of femoral artery. Surprisingly, just two out of five kidneys were Olfr78 positive.

Next, we used the mouse strain CD1;129-Olfr78^tm1jgst^ to clarify which cells of the organs/tissues express Olfr78.

### Differentiation between endogenous *β*-galactosidase activity and that resulting from lacZ gene expression

In the mouse strain CD1;129-Olfr78^tm1jgst^ green fluorescent protein (GFP) and tau/*β*-galactosidase are co-expressed with Olfr78. We decided to focus on *β*-galactosidase as histological marker because it enabled the analysis of both whole organs for Olfr78 expression and frozen sections generated from these organs.

**Fig. 2:**
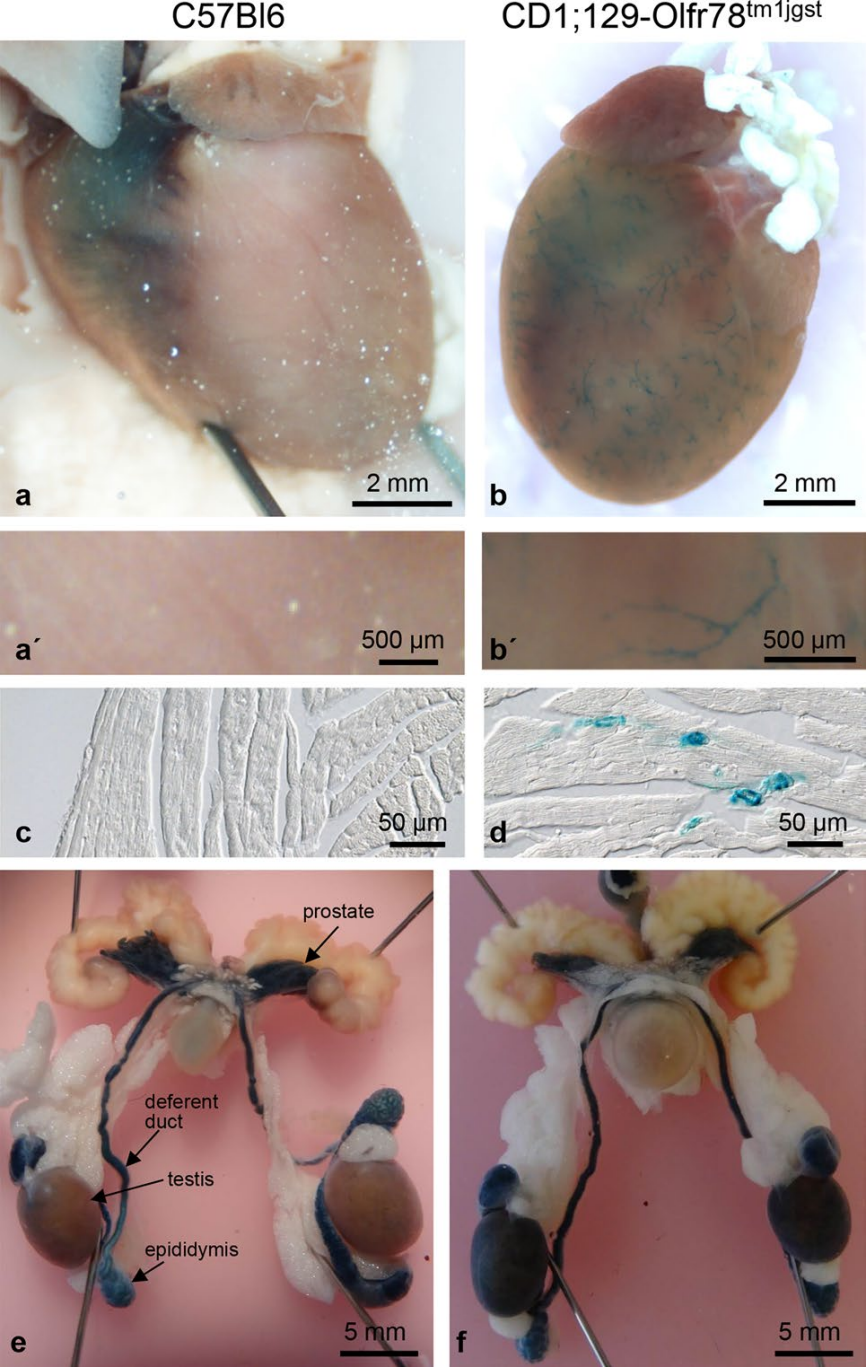
Differentiation between endogenous *β*-galactosidase activity and that resulting from tau-lacZ gene expression. X-gal staining of whole hearts (**a**, **b**) and corresponding frozen sections (**c**, **d**) of a nontransgenic C57Bl6 mouse (**a**, **c**) and of a CD1;129-Olfr78^tm1jgst^ mouse (= Olfr78 reporter mouse) (**b**, **d**). **aʹ**, **bʹ** details of the ventricle walls of whole hearts at higher magnification. Whole-mount X-gal staining of male reproductive tract of a nontransgenic C57Bl6 mouse (**e**) and an Olfr78 reporter mouse (**f**). Endogenous X-gal activity is detectable in epididymis, deferent ducts, and prostate, and staining of testis is restricted to the reporter mouse

X-gal is a commonly used substrate for the detection of bacterial *β*-galactosidase (lacZ). Most mammalian *β*-galactosidases have an acidic pH optimum whereas the bacterial X-gal enzyme works best at neutral pH. However, Weiss et al. (1999) have shown that at pH 7.5—which is recommended in most X-gal staining protocols and which we have used too—both endogenous and bacterial *β*-galactosidase may be active. Therefore, we compared X-gal staining of whole organs from C57Bl6 (Fig. 2a, aʹ, c, e) and Olfr78 reporter mice (CD1;129-Olfr78^tm1/gst^; Fig. 2b, bʹ, d, f). The heart of the C57Bl6 mouse was negative (Fig. 2a, aʹ), whereas in the heart of the reporter mouse (Fig. 2b, bʹ) X-gal reaction product was detectable in small vessels of the ventricles. Accordingly, X-gal-positive vessels were present in frozen sections of hearts from reporter mice, but not from C57Bl6 animals (Fig. 2 c, d). In the male reproductive tract, however, endogenous *β*-galactosidase activity resulted in staining of prostate, epididymis, and deferent duct of both mouse strains (Fig. 2 e, f). Staining of testis was restricted to the reporter mice indicating Olfr78 expression in this organ (Fig. 2f). In the following, we will present data only from findings in reporter mice.

In summary, most X-gal-positive cells were located in the walls of specific vascular beds. In particular, blood vessels supplying striated muscles of the musculoskeletal system and of the heart showed pronounced X-gal staining. Additionally, we observed staining of non-vascular cells. The expression of Olfr78 in non-sexual organs was comparable in male and female mice.

### Skeletal muscles are supplied with blood by vessels exhibiting Olfr78-expressing cells

**Fig. 3:**
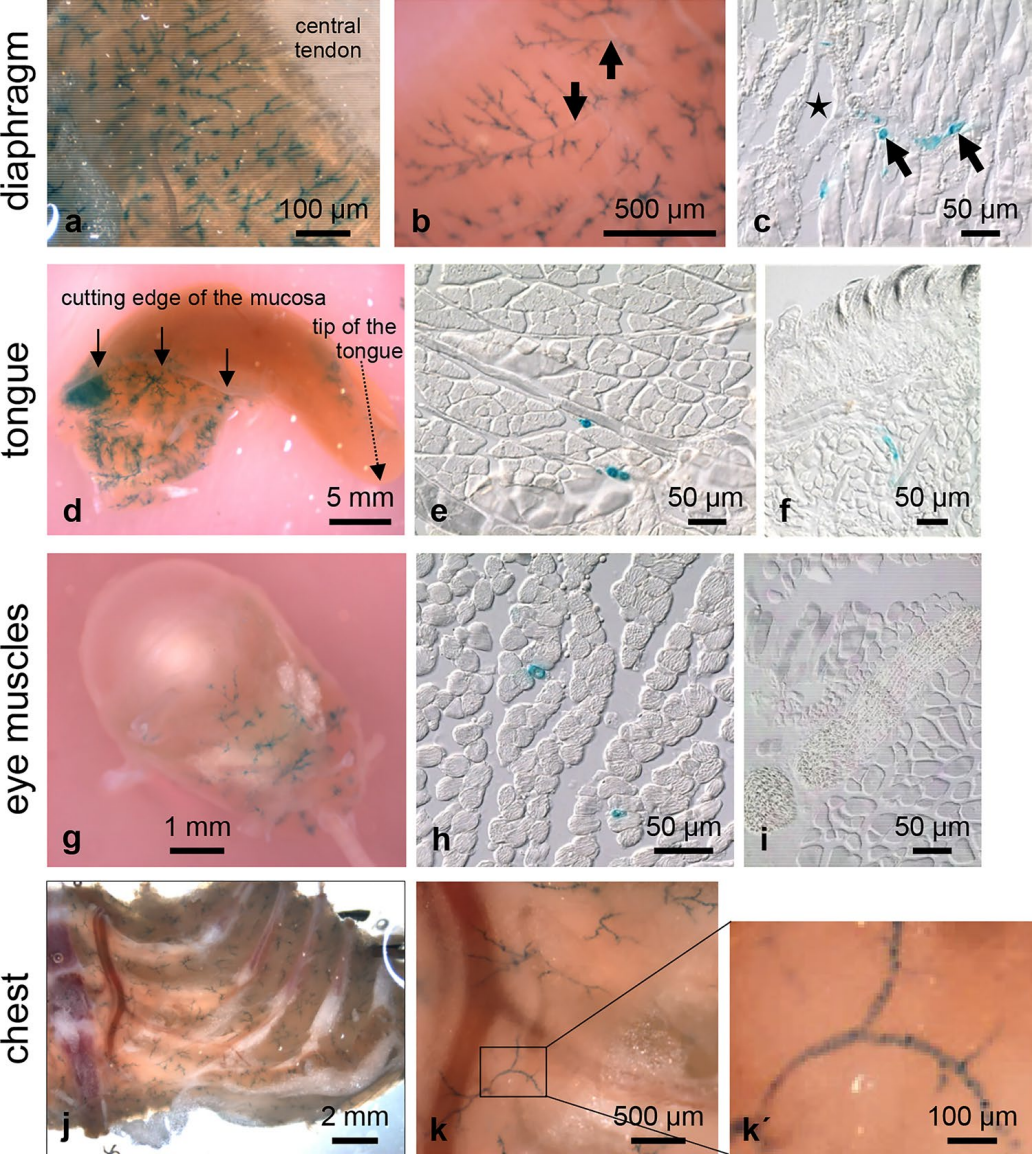
X-gal-positive small vessels supplying striated muscles of various regions of the mouse body. The muscle of the diaphragm, but not the central tendon, possesses X-gal-positive vessels (**a**). At higher magnification, strong X-gal staining of small vessels can be seen, whereas larger vessels (arrows) show weak or no labelling (**b**). Corresponding frozen section of the diaphragm showing a larger, negative vessel (asterisk) and small, positive vessels (arrows) (**c**). In the tongue, penetration of the solutions necessary for X-gal staining is restricted by the mucosa wrapping the muscular body. In the region of removed mucosa, a distinct X-gal labelling of small vessels supplying the skeletal muscle is obvious (**d**). Frozen section of the tongue with small X-gal-stained vessels (**e**). No X-gal-positive cells are detectable in the epithelium of the dorsum of the tongue (**f**). Whole-mount X-gal staining of the eye (**g**) reveals small positive vessels running in the extraocular muscles (clearing of the sample for 19 days); the corresponding frozen section is shown in (**h**). Nerve fibers running between the muscle fibers are negative (**i**). Whole-mount X-gal staining of the chest (**j**) demonstrates that small vessels supplying the intercostal muscles are X-gal positive, whereas the larger vessels are negative (**k**). The boxed area marked in (**k**) is given at higher magnification in (**kʹ**) to demonstrate the circular arrangement of the X-gal-positive cells in the vessel wall

**Fig. 4:**
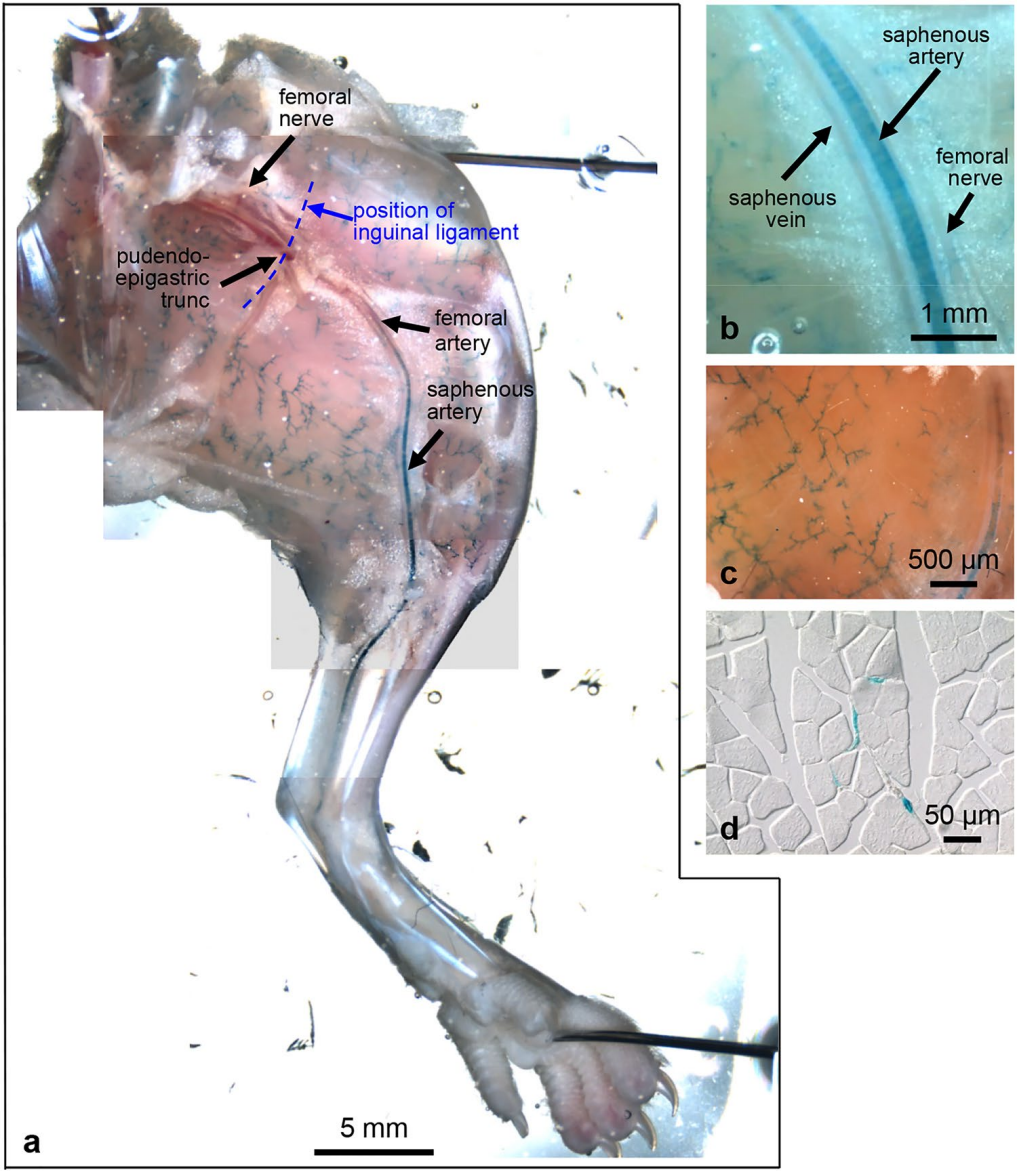
Large and small arteries of the hind limb exhibit X-gal-positive segments. Overview of whole-mount X-gal staining of part of the opened pelvis and the medial aspect of the left hind limb after removal of skin and fat tissue (**a**). The external iliac artery running in the pelvic is X-gal negative. After crossing the inguinal ligament (dashed blue line), the vessel is called the femoral artery. It becomes X-gal positive in its course to the thigh. When the femoral artery continues into the saphenous artery, it exhibits continuously increasing X-gal staining intensity. Higher magnification reveals the circular arrangement of X-gal-stained cells of the arterial wall. The accompanying saphenous vein and femoral nerve are unstained (**b**). Small vessels supplying the skeletal muscles show strong X-gal staining (**c**) as can also be seen in the corresponding frozen section (**d**)

Pluznick et al. have described the expression of Olfr78 in small resistance vessels supplying the diaphragm and some non-specified skeletal muscles (Pluznick et al. 2013). We confirmed and extended these findings by analysing the diaphragm and defined skeletal muscles taken from all major regions of the body (Figs. 3 and 4). In the diaphragm, X-gal-positive vessels were arranged in a dense network, which was detectable in both whole-mount samples and frozen sections (Fig. 3a–c). We also found X-gal staining in small vessels feeding the intrinsic muscles of the tongue (Fig. 3d–f) and the extrinsic muscles of the eyeball (Fig. 3g–i). Nerve fibres supplying these striated muscles (Fig. 3i) and the optic nerve (Fig. 3g) were X-gal negative. The microvasculature of intercostal muscles of the chest (Fig. 3j, k, kʹ), thigh muscles (Fig. 4a, c, d), and muscles of the pelvic floor (Fig. 7i) was X-gal positive, too. Furthermore, we observed X-gal-stained vessels in facial muscles at the tip of the nose, cheek muscles, and muscles of the floor of the mouth (not shown). The outer diameter of most of these intensively stained small vessels was in the range of 6 to 12 µm.

With exception of the hind limbs, staining was restricted to the microvasculature whereas large(r) vessels were unlabelled. The internal thoracic artery and the intercostal arteries were unlabelled whereas the small vessels running in the intercostal muscles exhibited distinct staining (Fig. 3j, k). Branches arising from these small vessels rapidly lost their labelling (Fig. 3k, k´), indicating that Olfr78 expression is restricted to very specific vascular segments.

The vasculature supplying the skeletal muscles of the hind limbs showed a distinct pattern of Olfr78 distribution in that large vessels were stained in this body region. Interestingly, occurrence of Olfr78 reporter-positive cells changed along the course of the vessel from the pelvis towards the hind paw: the main feeding artery starting in the pelvis—the external iliac artery—was completely negative (Fig. 4a). X-gal staining of the neighbouring small vessels running in the muscles indicates that this was not due to insufficient penetration with reaction solution (Fig. 4a, c). After undercrossing the inguinal ligament, the vessel—now called femoral artery—became increasingly X-gal positive (Fig. 4a, b). When it crossed the distal thigh and became the saphenous artery, intense labelling became obvious, which was detectable until the X-gal signal disappeared in the region of the lower leg (Fig. 4a). Vascular staining was not homogeneous: it was caused by X-gal-positive cells circularly arranged in the arterial wall (Fig. 4b). Along the entire course of the artery, the accompanying vein and nerve were negative (Fig. 4b). As in skeletal muscles of other regions, small X-gal-positive vessels formed a network within the musculature that was also detectable in frozen sections (Fig. 4d).

**Fig. 5:**
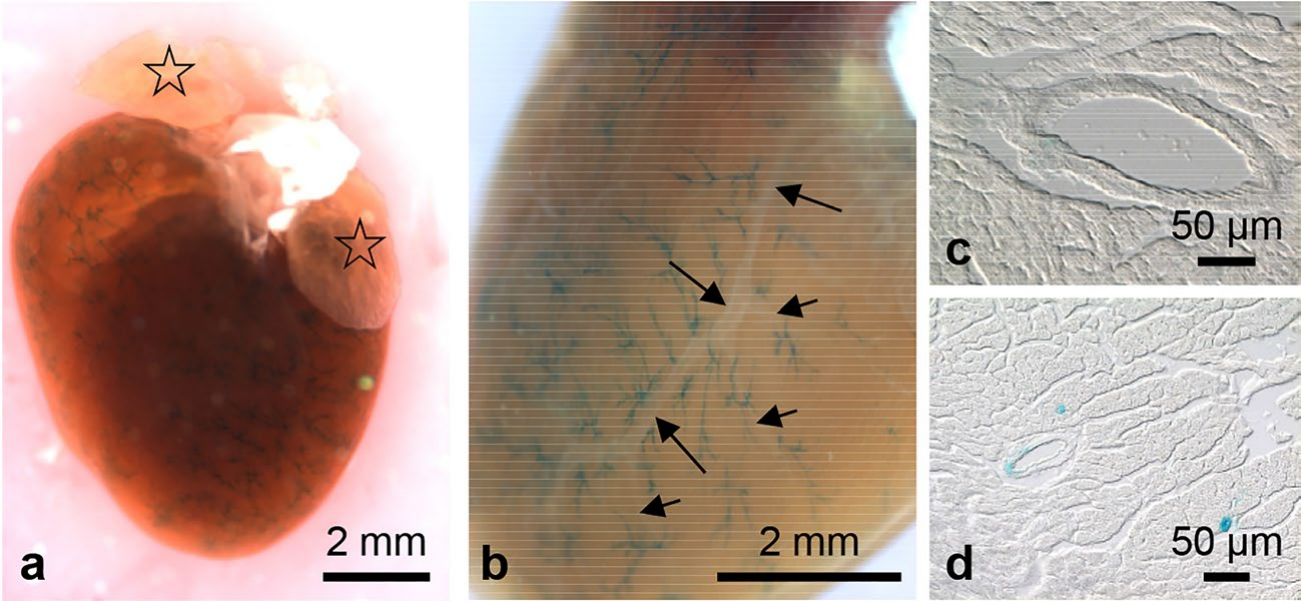
X-gal staining of the heart. Whole-mount heart cleared for 10 days with intensively stained small vessels (**a**). Heart ventricles exhibit a dense network of X-gal-positive small vessels, whereas atria are almost negative (asterisks in **a**). Major coronary arteries (long arrows) and their main branches (short arrows) are X-gal negative (**b**). Frozen sections showing a large, X-gal-negative (**c**) and small, X-gal-positive vessel (**d**)

X-gal staining was not restricted to small vessels feeding the striated muscles of the musculoskeletal system, but also detectable in the vessels supplying the striated muscles of the heart (Figs. 2 and 5). In cleared hearts, the network of X-gal-positive vessels was particularly well recognizable in the ventricles (Fig. 5a, b) but hardly detectable in the atria (Fig. 5a). In the heart, X-gal-positive vessels reached diameters up to 30 µm. In contrast to these small blood vessels (Fig. 5b, d), the large vessels feeding this network were X-gal negative (Fig. 5b) as could also be seen in frozen sections (Fig. 5c).

### Organ-specific differences in the contribution of Olfr78 reporter-expressing vessels to the blood supply

**Fig. 6:**
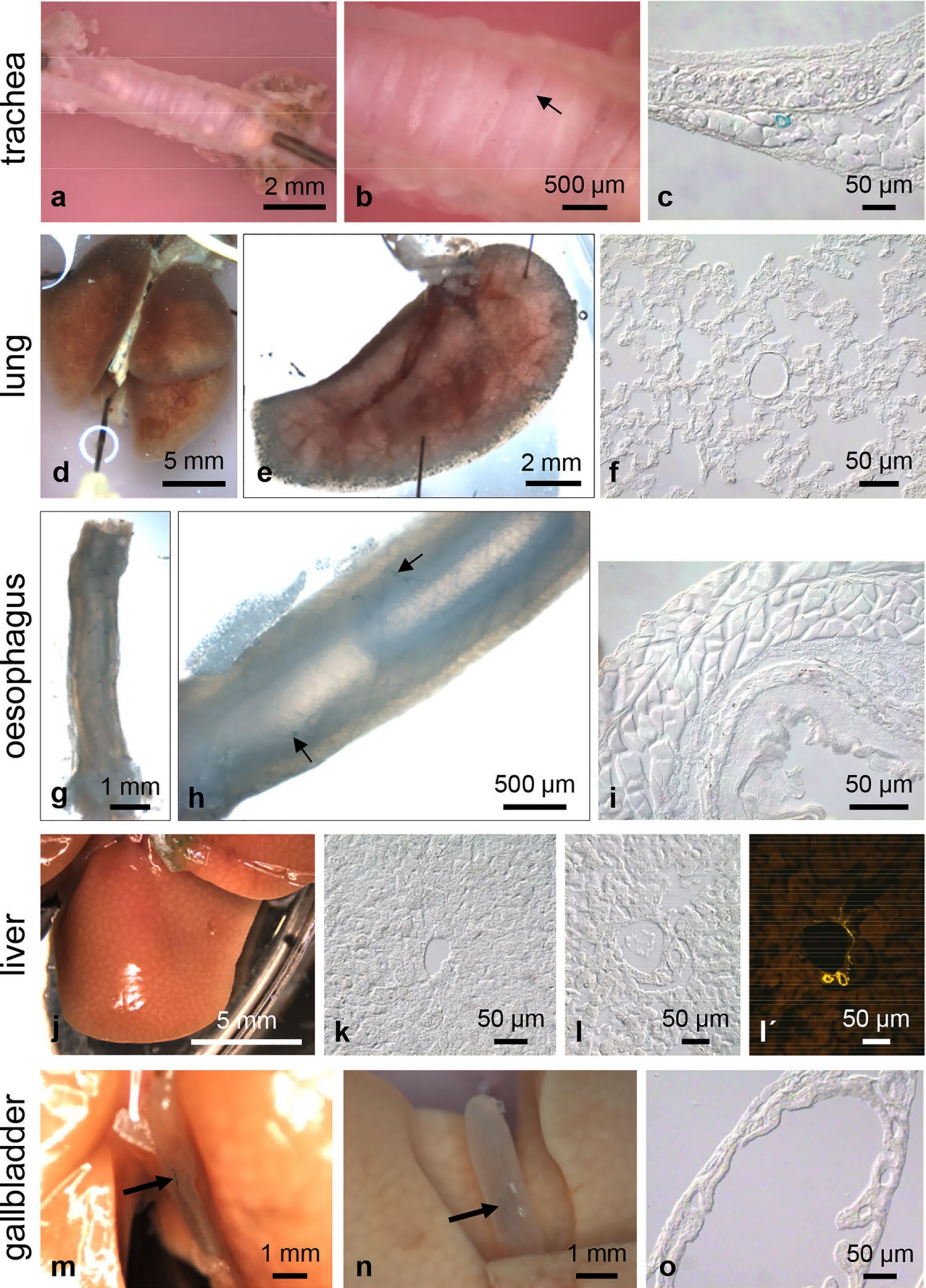
Whole-mount X-gal staining of respiratory and gastrointestinal organs. In the longitudinally opened trachea (**a**), X-gal-positive cells are hardly detectable. An individual small vessel with X-gal-positive cells (arrow) can be seen at higher magnification in (**b**). Corresponding frozen section with a small X-gal-positive vessel in the tracheal adventitia (**c**). X-gal staining cannot be seen in the overview of both lungs (**d**), at higher magnification of one lobe (**e**), or in frozen sections (**f**). In the oesophagus, only a few small X-gal-positive vessels are detectable (**g**, arrows in **h**). Corresponding frozen section without X-gal reaction product (**i**). Whole-mount stained liver (**j**) and corresponding frozen sections (**k**, **l**) are completely X-gal negative. Smooth muscle actin immunohistochemistry with a Cy3-conjugated antibody was performed to visualize the branches of the hepatic artery and portal vein within Glisson’s triad. None of the vessels were X-gal positive (**l**, **lʹ**). In gallbladders of two mice, only weak staining of the cystic artery can be seen (**m**, **n**). Corresponding frozen section (**o**)

**Fig. 7:**
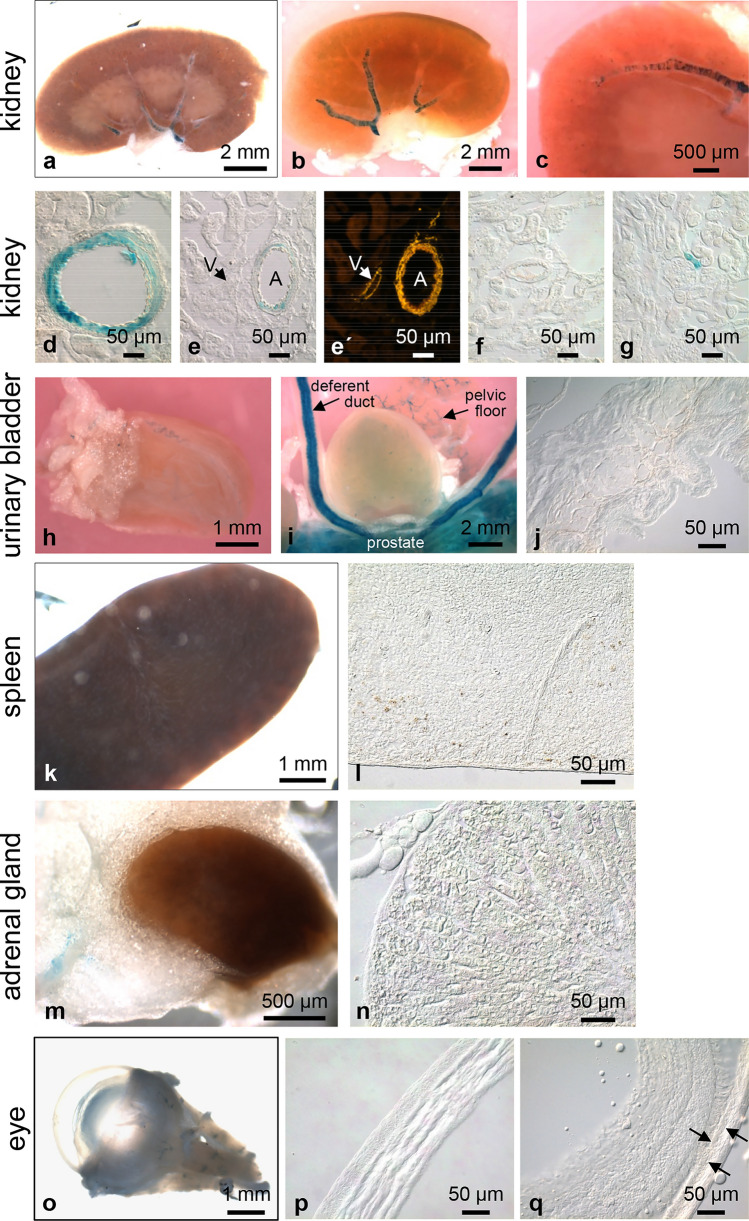
Whole-mount X-gal staining of murine organs. Kidney is the only region of the murine body other than the hind limb in which we observed larger X-gal-positive vessels (**a**). In a kidney cleared for 19 days (**b**), positive vessels enter at the hilum. Higher magnification reveals the circular arrangement of the X-gal-positive cells (**c**). The frequency of such cells in the vessel wall decreases towards the periphery. Frozen sections demonstrate closely packed X-gal-positive cells in a large vessel (**d**), a loose arrangement in a medium-sized vessel (**e**), absence in a small vessel (**f**), and occurrence in the afferent arteriole of a renal corpuscle (**g**). X-gal-positive cells are located in the wall of arteries (A), but not of veins (V); smooth muscle cells labelled with Cy3-conjugated smooth muscle actin antibody (**e**, **eʹ**). In the urinary bladder, X-gal-positive vessels can be found only sporadically (**h**, **i**). In the vicinity of the urinary bladder, remnants of the pelvic floor muscle with small X-gal-positive vessels and parts of the strongly stained deferent duct and prostate can be seen (**i**). Corresponding frozen section of the urinary bladder (**j**). Whole mounts of spleen (**k**) and adrenal gland (**m**) are X-gal negative as can also be seen in the corresponding frozen sections (**l**, **n**). In the eye (**o**), apart from the extraocular muscles, no X-gal-positive structures are detectable. Frozen sections from cornea (**p**) and retina (**q**) are X-gal negative. Arrows mark the retinal pigmentary epithelium

**Fig. 8:**
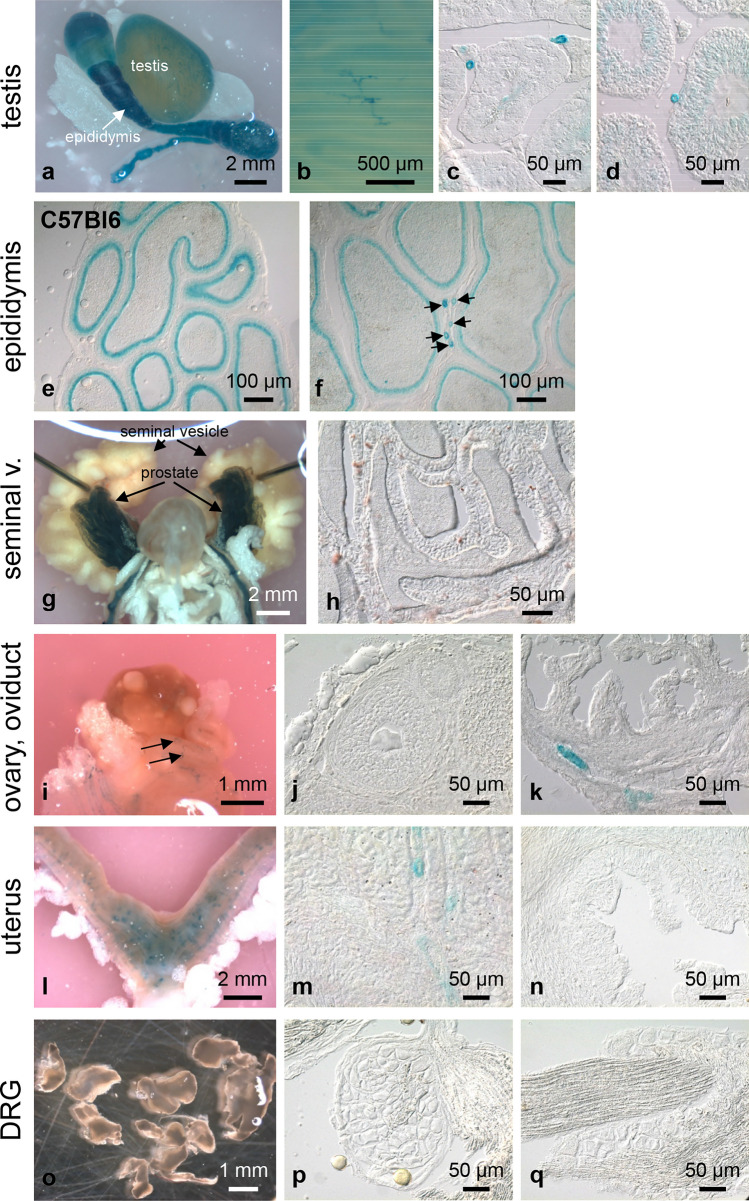
Whole-mount X-gal staining of genital organs and DRG. In whole-mount testis cleared for 12 days, X-gal-stained seminiferous tubules and small vessels can be identified (**a**, **b**). In frozen sections, X-gal staining of maturing sperm can be seen. Staining intensity changes in the different development phases. Small vessels running between the tubules are strongly X-gal positive (**c**, **d**). In the epididymis, small vessels are X-gal positive (arrows) (**f**). Staining of the epididymal epithelium is caused by endogeonous X-gal activity as can be seen in frozen sections of WT epididymis (**e**). The seminal vesicles (seminal v.) are completely X-gal negative (**g**). Corresponding frozen section (**h**). In the female reproductive tract, the ovary is X-gal negative (**i**, **j**). On the surface of the oviduct (arrows) and in the surrounding adipose tissue, small X-gal-positive vessels are detectable (**i**). Frozen section of the oviduct with X-gal-positive vessels (**k**). The uterus—especially the fundus—shows a relatively high density of X-gal-positive vessels (**l**), which can also be seen in frozen sections (**m**). The epithelium of the uterus is X-gal negative (**n**). No X-gal staining is detectable in DRG and spinal nerve (**o**–**q**)

**Fig. 9:**
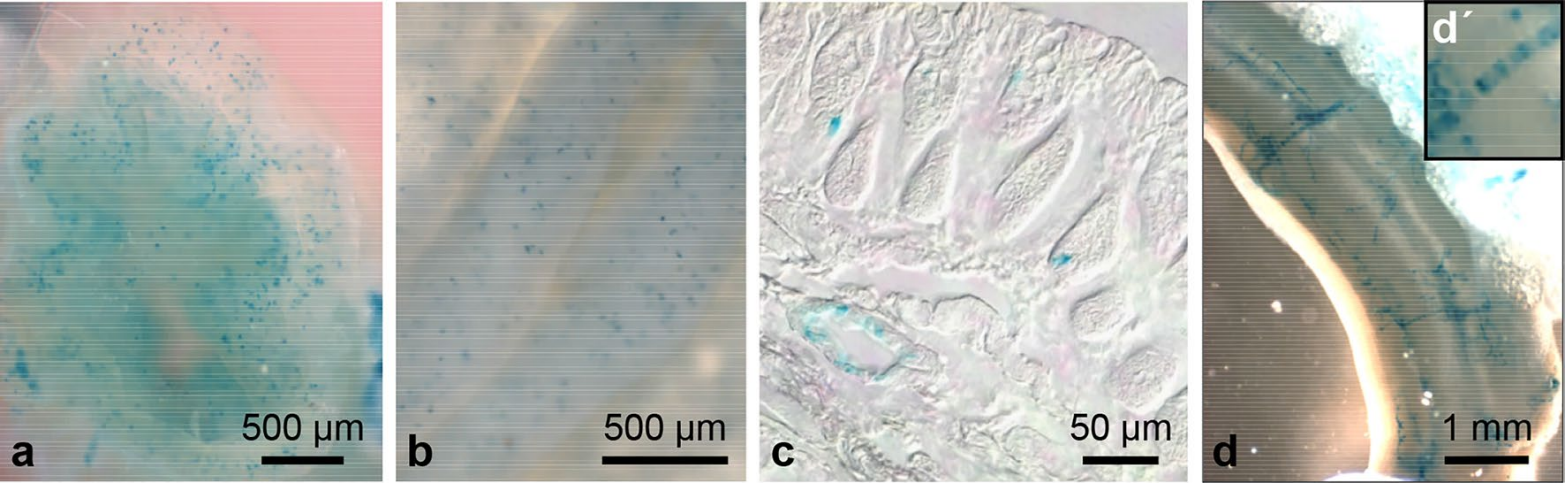
X-gal staining of the colon. View on a cross-sectioned whole-mount colon after clearing for 7 days with dotted X-gal staining pattern of the mucosa (**a**), which becomes more apparent in a longitudinally opened colon (**b**). Frozen section showing individual X-gal-positive cells in the colonic epithelium and a small X-gal-positive vessel (**c**). X-gal-stained vessels running on the outer face of the whole-mount colon are shown in (**d**). At higher magnification (**dʹ**) the circular arrangement of the X-gal-positive cells can be seen

Additionally, we screened many murine organs for X-gal expression; these findings are compiled in Figs. 6, 7, 8, 9. In summary, most of the X-gal-positive cells were located in vessels supplying the organs with blood.

Vascular LacZ expression differed distinctly between organs. We did not detect vessels with X-gal-positive cells in lung (Fig. 6d–f), liver (Fig. 6j–l), adrenal gland (Fig. 7m, n), spleen (Fig. 7k, l), seminal vesicle (Fig. 8g, h), ovary (Fig. 8i, j), dorsal root ganglia (DRGs), and spinal nerves (Fig. 8o–q). In the urinary bladder, X-gal-positive small vessels were so rare that we detected them in whole mounts of the organs but not in frozen sections (Fig. 7h–j). In trachea (Fig. 6a–c) and the oesophagus (Fig. 6g, h) only individual small vessels were X-gal positive. In the gallbladder, we regularly observed staining of the cystic artery, but to a variable degree between animals (Fig. 6m, n). Vessels exhibiting X-gal-positive cells were consistently found in colon (Fig. 9c, d), kidney (Fig. 7a–g), testis (Fig. 8a–d), epididymis (Fig. 8f), oviduct (Fig. 8i, k), and uterus (Fig. 8l, m). X-gal staining, however, was not continuous along the arterial supply in any of these organs. In colonic vessels, cells of different staining intensities including negative cells alternated in an irregular order (Fig. 9dʹ).

In the kidney, the various segments of vessels had different staining patterns (Fig. 7a–g). X-gal-positive cells were restricted to arteries and not detectable in accompanying veins (Fig. 7e, eʹ). The main branches of the renal artery (external diameter about 200 µm) were covered by circularly arranged positive cells. The density of these cells rapidly decreased on the way of the vessel from the hilum to the cortex (Fig. 7b, c). However, the vascular section in which loss of X-gal-positive cells occurred varied between individual animals ranging from relatively near to the hilum to somewhere in the cortex region. The following vessel segment (medium-sized vessels with external diameters between 100 and 200 µm) had no positive or just a few positive cells (Fig. 7e). Smaller vessels with diameter between 30 to 100 µm were mostly negative (Fig. 7f). Some of the small vessels in close proximity of glomeruli (outer diameter between 7 and 11 µm)—probably afferent arterioles—showed strong staining.

In the testis, small vessels running between the seminiferous tubules (Fig. 8c, d) were strongly stained. This network of vessels was already visible from the outer aspect of the intact testis (Fig. 8a, b). In the female reproductive tract, the ovary was found to be X-gal negative (Fig. 8i, j). In the oviduct, we observed small X-gal-positive vessels in both the whole organ and frozen sections (Fig. 8i, k). The uterine fundus showed a relatively high density of X-gal-positive vessels (Fig. 8l) and in frozen sections these stained vessels were detectable in the myometrium (Fig. 8m); the epithelium exhibited no X-gal-positive cells (Fig. 8n).

### Olfr78 reporter-expressing cells in the vessel wall are smooth muscle cells

Frozen sections of kidney, heart ventricles, and testis with X-gal-positive vessels were labelled with FITC- or Cy3-conjugated anti-α-smooth muscle actin antibody to identify smooth muscle cells, revealing colocalization of the signals (Figs. 7e, eʹ and 10).

**Fig. 10:**
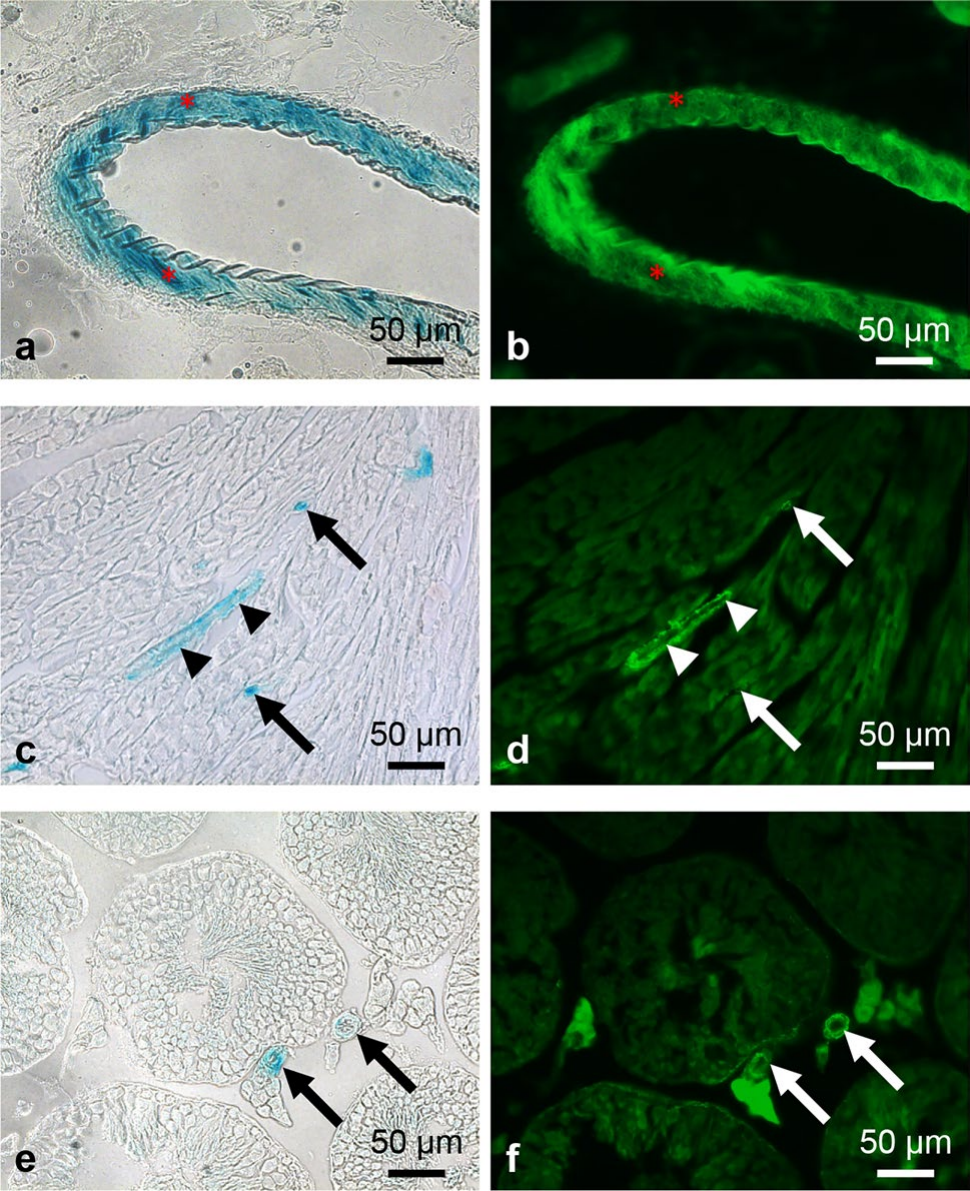
X-gal-positive vascular cells are smooth muscle cells. Frozen sections of whole-mount X-gal-stained kidney (**a**, **b**), heart (**c**, **d**), and testis (**e**, **f**) were stained with a FITC-conjugated anti-smooth muscle actin antibody to visualize smooth muscle cells. X-gal reaction product and fluorescence signal are colocalized, indicating Olfr78 expression by smooth muscle cells. However, large amounts of X-gal reaction product prevent binding of the antibody (asterisks in **a**, **b**). Arrowheads in (**c**, **d**) point to a longitudinally sectioned small vessel, arrows in (**c**–**f**) to cross sectioned small vessels

### Non-vascular Olfr78 expression

**Fig. 11:**
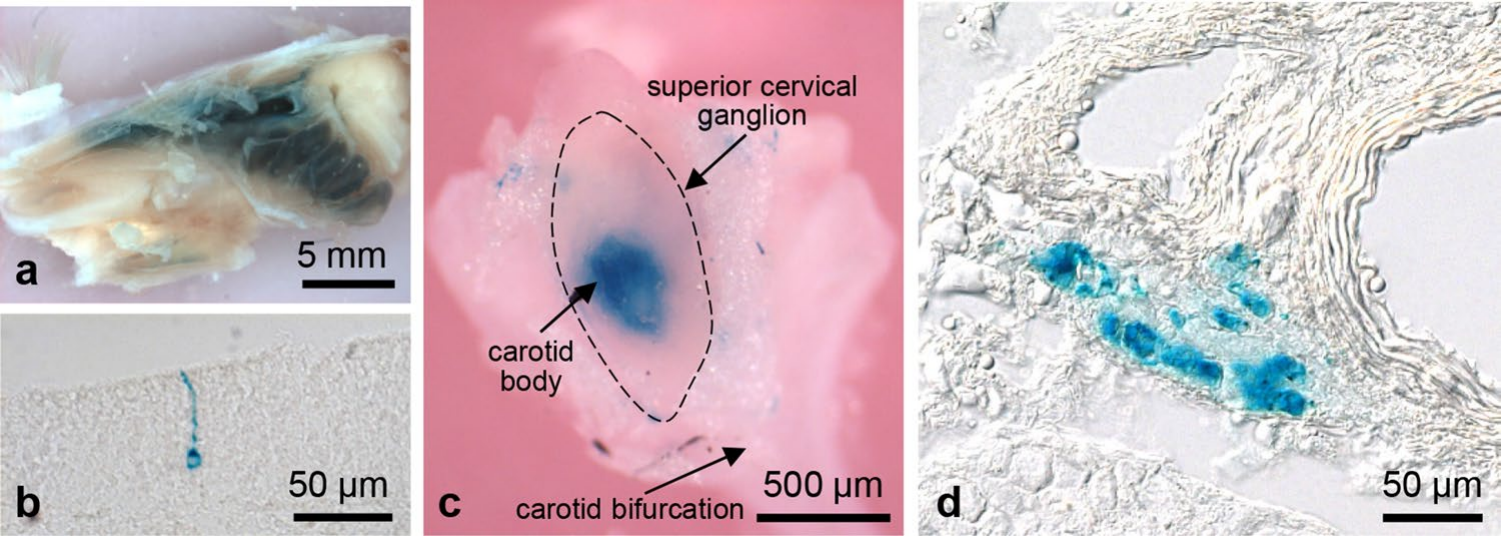
X-gal staining of the olfactory epithelium and carotid body. X-gal staining of the nasal cavity with intensively stained olfactory epithelium (**a**). In the frozen section (**b**), a single positive olfactory neuron is shown. X-gal staining of the carotid body attached to the superior cervical ganglion (bordered by a dashed line) of the sympathetic trunk, which is located in the carotid bifurcation (**c**). The tissue was cleared for 10 days. Corresponding frozen section of the carotid body with intensively stained glomus cells (**d**)

Abundant Olfr78 expression has been reported previously for the olfactory epithelium and carotid body (Conzelmann et al. 2000; Chang et al. 2015; Zhou et al. 2016). In agreement with Conzelmann et al. (2000), we observed strong X-gal staining of the olfactory epithelium in whole mounts of the nasal cavity (Fig. 11a) and detected individual X-gal-positive olfactory neurons in corresponding frozen sections (Fig. 11b). In the carotid body, in addition to the previously reported staining of glomus cells, we observed scattered X-gal-positive small vessels running in the adipose tissue surrounding the carotid body and superior cervical ganglion, whereas the large arteries of the carotid bifurcation were unlabelled (Fig. 11c, d). Fleischer et al. (2015) have described Olfr78 expression by a subset of epithelial cells lining the crypts of the colon and identified these cells as enteroendocrine cells. In line with this report, we also observed numerous X-gal-positive cells interspersed among the mucosal epithelium in colonic whole mounts (Fig. 9a, b) and sectioned colonic crypts (Fig. 9c). In testis, we observed a weak staining of maturing sperm (Fig. 8c, d). Differences in the intensity of the labelling probably reflect different stages of development of the sperm. Xu et al. (2000) have performed in situ RNA hybridization to demonstrate Olfr78 expression in prostate tissues with a predominant localization in epithelial cells. Due to the high endogenous X-gal activity of prostate (Figs. 2e, 8g), we cannot draw conclusions about Olfr78 expression in this organ (Fig. 2e). We detected Olfr78 expression in neither the retinal pigment cells and choroid as described by Jovancevic et al. (2017) for the human eye (Fig. 7q) nor nervous elements in DRGs, superior cervical ganglion, peripheral nerves, enteric plexuses, and nerve fibres within the organs investigated. Findings obtained by X-gal staining are summarized in Table 1.

## Discussion

Recently, more and more studies have demonstrated distinct functions of ectopically expressed ORs (Maßberg and Hatt 2018; An and Liggett 2018). Detection of OR transcript is mostly based on RT-PCR, microarray or RNA sequencing techniques. Yet, generation of specific antibodies is difficult since members of OR families have > 40% sequence identity. The olfactory receptor family 51 (OR51), to which Olfr78 belongs, includes 44 members (“HUGO Gene Nomenclature Committee at the European Bioinformatics Institute”; https://www.genenames.org/data/genegroup/#!/group/164). Therefore, we have used transgenic mice expressing the lacZ reporter under the control of the Olfr78 promotor to detect Olfr78-expressing cells in many organs and tissues.

We found that in many Olfr78 mRNA-expressing organs, X-gal product was localized in small vessels within the organ and/or running in the surrounding adipose tissue, but not in the parenchyma or in connective tissue cells of the stroma of the organ. The only exceptions that we found were glomus cells of the carotid body, sensory cells of the olfactory mucosa, enteroendocrine cells of the colon, and maturing sperm in the testis. These findings are consistent with data published by Conzelmann et al. (2000), Chang et al. (2015), Fleischer et al. (2015), Torres-Torrelo et al. (2018), and Flegel et al. (2016). However, we could not confirm expression of Olfr78 in retinal pigment cells and the choroid as described by Jovancevic et al. (2017) for the human eye. Contrary to the findings of Pluznick et al. (2013), we found no labelling of axons of the autonomic nervous system in heart, oesophagus, stomach, and other structures of the nervous system (DRGs, the femoral nerve, nerves supplying the extraocular muscles were X-gal negative). Whereas expression of OR51E2 by cultured primary human airway smooth muscle cells has been demonstrated by RT-PCR (Natarajan et al. 2016), we obtained an Olfr78 RT-PCR product in just two of five lung samples and detected no X-gal staining in whole lungs or frozen sections of the organ. In all other organs in which we detected Olfr78 mRNA, the X-gal reaction product was localized in vascular smooth muscles cells, mostly of arterioles. We found no correlation between organs with X-gal-positive vessels and organ systems. In the female genital tract, for example, the ovary was negative and oviduct and uterus were positive. In the digestive tract, the esophagus was almost negative and the colon showed X-gal-positive vessels.

The localization of Olfr78 in smooth muscle cells of the resistance vessels of the heart, diaphragm, skeletal muscle, and skin as well as in vascular smooth muscle cells of afferent arteriole of the juxtaglomerular apparatus of the kidney led Pluznick et al. (2013) to suspect that Olfr78 plays a role in blood pressure regulation. Subsequent experiments revealed that blood pressure regulation by SCFA, ligands of Olfr78, include another GPCR, Gpr41 (synonym: free fatty acid receptor 3). Using a luciferase-based reporter assay in which OR-ligand binding produces an increase in cAMP that drives cAMP response element-dependent expression of luciferase, Pluznick et al. (2013) have demonstrated that Gpr41 is activated by fairly low concentrations of propionate (EC_50_ =  ∼ 11 μM) whereas Olfr78 responds to high concentrations of propionate (EC_50_ =  ∼ 0.9 mM). In mice, activation of Gpr41 by SCFAs lowers the blood pressure, whereas Olfr78 counters this response. Pluznick’s group postulated that Olfr78 may act as a safety “brake” on Gpr41-mediated drops in blood pressure preventing an inappropriate level of hypotension, for example after a meal when blood concentration of SCFA is increased (Pluznick et al. 2013; Natarajan et al. 2016; Pluznick 2017). Such a mechanism seems particularly useful for the digestive tract to optimize the absorption of nutrients into the bloodstream through vasodilation without reducing blood pressure to critical levels.

Remarkably, we found Olfr78 expression especially in small blood vessels supplying striated—cardiac and skeletal—muscles widely distributed over the mouse body, independent of embryonic origin of the muscle fibres and their vascular and connective tissue elements. In craniofacial muscles, such as cheek muscles, the muscle fibres are derived from cranial paraxial and prechordal head mesoderm and the connective tissue with its vessels from the neural crest (Noden and Francis-West 2006; Ziermann et al. 2018). Myogenic cells of trunk muscles, such as intercostal muscle, derive from the epaxial lip and diaphragm and limb muscles from the hypaxial lip of the dermomyotome, with connective tissue coming from somites in the trunk and from the lateral plate mesoderm in the limbs (Nassari et al. 2017). Indicated by the X-gal product, Olfr78 expression was localized in circularly arranged smooth muscle cells. We identified these vessel segments as arterioles based on the vessel diameter, thickness of the muscle layer, and density of the smooth muscle cells. In agreement with Pluznick et al. (2013) we found, that only a subset of anti-α-smooth muscle actin-positive cells of the muscle layer showed the X-gal product.

Since each regional circulation of the body has unique requirements for blood supply and thus unique mechanisms for blood flow regulation, also the function/regulation of Olfr78 may differ regionally (Reho et al. 2014). Contraction of the skeletal muscles results in marked dilation in all elements of the arteriolar tree, with the most pronounced dilation in the smallest arterioles (Joyner and Casey 2015). A number of vasodilators are formed in exercising muscles and it appears that none of them operate independently or are essential for reaching adequate blood flow during exercise. This redundancy is an important concept to secure exercise hyperaemia for adequate oxygen supply (Hellsten et al. 2012). Bradykinin is a potent vasodilator that acts through bradykinin receptor 2 (B2R), which is constitutively expressed by endothelial cells. Ligand binding causes the release of nitric oxide and endothelium-derived hyperpolarizing factor resulting in relaxation of smooth muscles cells. Recently, bradykinin was identified as a novel agonist of OR51E2 (Abaffy et al. 2018). Using a luciferase-based reporter assay Abaffy et al. estimated an EC_50_ value of 1.30^–9^ M (= 1.3 nM) for bradykinin. The median bradykinin concentration in plasma samples from healthy volunteers is 0.7 nmol/l (range 0.5–1.1 nmol/l) (Lindström et al. 2019). Already in 1990, Stebbins et al. (1990) described that in cats electrical stimulation of the triceps surae muscles causes a distinct increase in bradykinin concentration in the venous effluent of the muscle. Langberg et al. (2002) reported an interstitial concentration of bradykinin in the human medial gastrocnemius muscle of 23.1 nmol/l, which raises to 110.5 nmol/l in response to exercise. In principle, these bradykinin concentrations would be sufficient to activate Olfr78 expressed by vascular smooth muscle cells of the skeletal microvasculature and probably contribute to exercise induced hyperaemia. The high expression of Olfr78 in the smooth muscle cells of the femoral artery may also participate in regulation of the blood flow to the muscles of the limb.

If Olfr78 is to be used as a target for the development of new therapies of hypertension or of prostate cancer, the widespread expression of the receptor in small blood vessels especially of striated muscles must be kept in mind.

## Data Availability

The data that support the findings of this study are available from the corresponding author upon reasonable request.
